# An integrated infant and young child feeding and small‐quantity lipid‐based nutrient supplementation programme in the Democratic Republic of Congo is associated with improvements in breastfeeding and handwashing behaviours but not dietary diversity

**DOI:** 10.1111/mcn.12784

**Published:** 2019-02-27

**Authors:** Lindsey M. Locks, Simeon Nanama, O. Yaw Addo, Bope Albert, Fanny Sandalinas, Ambroise Nanema, Ralph D. Whitehead, Aashima Garg, Roland Kupka, Maria Elena Jefferds, Katie Tripp

**Affiliations:** ^1^ Department of Nutrition Harvard T.H. Chan School of Public Health Boston MA USA; ^2^ Programme Division United Nations Children's Emergency Fund (UNICEF) Headquarters New York NY USA; ^3^ Programme Division United Nations Children's Emergency Fund (UNICEF) Kinshasa and Lubumbashi Democratic Republic of Congo; ^4^ Rollins School of Public Health Emory University Atlanta GA USA; ^5^ International Micronutrient Malnutrition Prevention and Control (IMMPaCt) Program U.S. Centers for Disease Control and Prevention Atlanta GA USA; ^6^ National Statistics Institute Lubumbashi Democratic Republic of Congo; ^7^ West and Central Africa Regional Office United Nations Children's Emergency Fund (UNICEF) Dakar Senegal

**Keywords:** breastfeeding, community‐based, complementary foods, International Child Health Nutrition, undernutrition, UNICEF

## Abstract

Integrating small‐quantity lipid‐based nutrient supplements (SQ‐LNS) into infant and young child feeding (IYCF) programmes can increase consumption of essential nutrients among children in vulnerable populations; however, few studies have assessed the impact of integrated IYCF–SQ‐LNS programmes on IYCF practices. A 2‐year, enhanced IYCF intervention targeting pregnant women and infants (0–12 months) was implemented in a health zone in the Democratic Republic of Congo (DRC). The enhanced IYCF intervention included community‐ and facility‐based counselling for mothers on handwashing, SQ‐LNS, and IYCF practices, plus monthly SQ‐LNS distributions for children 6–12 months; a control zone received the national IYCF programme (facility‐based IYCF counselling with no SQ‐LNS distributions). Cross‐sectional preintervention and postintervention surveys (*n* = 650 and 638 in intervention and control areas at baseline; *n* = 654 and 653 in each area at endline, respectively) were conducted in mothers of children 6–18 months representative of both zones. Difference in differences (DiD) analyses used mixed linear regression models. There were significantly greater increases in the proportion of mothers in the intervention (vs. control) zone who reported: initiating breastfeeding within 1 hr of birth (Adj. DiD [95% CI]: +56.4% [49.3, 63.4], *P* < 0.001), waiting until 6 months to introduce water (+66.9% [60.6, 73.2], *P* < 0.001) and complementary foods (+56.4% [49.3, 63.4], *P* < 0.001), feeding the minimum meal frequency the previous day (+9.2% [2.7, 15.7], *P* = 0.005); feeding the child in a separate bowl (+9.7% [2.2, 17.2], *P* = 0.01); awareness of anaemia (+16.9% [10.4, 23.3], *P* < 0.001); owning soap (+14.9% [8.3, 21.5], *P* < 0.001); and washing hands after defecating and before cooking and feeding the child the previous day (+10.5% [5.8, 15.2], +12.5% [9.3, 15.6] and +15.0% [11.2, 18.8], respectively, *P* < 0.001 for all). The enhanced IYCF intervention in the DRC was associated with an improvement in several important IYCF practices but was not associated with a change in dietary diversity (minimum dietary diversity and minimum acceptable diet remained below 10% in both zones without significant differences between zones). The provision of fortified complementary foods, such as SQ‐LNS, may be an important source of micronutrients and macronutrients for young children in areas with high rates of poverty and limited access to diverse foods. Future research should verify the potential of integrated IYCF–SQ‐LNS to improve IYCF practices, and ultimately children's nutritional status.

**Key messages**

- The enhanced IYCF intervention in the DRC was associated with an increase in the prevalence of several important handwashing and breastfeeding practices, but there were no changes in dietary diversity among infants in this food insecure environment.
- Fortified foods may fill an important nutrient gap in areas with high rates of poverty and food insecurity; however, future research must assess whether integrated IYCF–SQ‐LNS programmes or programmes providing other micronutrient‐rich food supplements can improve children's nutritional status.
- Mothers in the intervention area were substantially more likely to report receiving IYCF messages from community health workers (CHWs) than mothers in the control area.
- CHWs may be an underutilized platform for supporting IYCF programmes in the DRC and elsewhere sub‐Saharan Africa; future research should focus on the cost and feasibility of expanding the use of CHWs for IYCF programmes.

**Abbreviations**
CHW: community health worker
DiD: difference in differences
IYCF: infant and young child feeding
MAD: minimum acceptable diet
MDD: minimum dietary diversity
MMF: minimum meal frequency
PR: prevalence ratio
SQ‐LNS: small‐quantity lipid‐based nutrient supplements

## 1 INTRODUCTION

Malnutrition during the critical period from conception to 2 years of age increases the risk of premature death and has lifelong developmental and health consequences (Dewey & Brown, 2003). Optimal infant and young child feeding (IYCF) practices—including initiation of breastfeeding in the first hour of birth, exclusive breastfeeding for the first 6 months of life, and the introduction of micronutrient‐rich, age‐appropriate foods from 6 months—are particularly important for ensuring the healthy growth and development of young children (Disha, Rawat, Subandoro, & Menon, 2012; Dykes & Hall‐Moran, 2009; Jones et al., 2014; Menon, Bamezai, Subandoro, Ayoya, & Aguayo, 2015; PAHO/WHO, 2003). In 2010, the United Nations Children's Emergency Fund (UNICEF) introduced the community‐based IYCF counselling package, which includes several planning, training, and counselling tools to support programming and capacity development on community‐based IYCF counselling (UNICEF, 2010). As of 2015, 60 countries had incorporated elements of the package; however, there has been little research assessing the use of this package at scale (Lamstein, 2017).

In settings with high rates of poverty and limited access to diverse diets, the distribution of nutritional supplements that are rich in micronutrients and macronutrients may improve IYCF practices and ultimately children's nutritional status (Dewey & Adu‐Afarwuah, 2008). Small‐quantity lipid‐based nutrient supplements (SQ‐LNS) are sachets containing ≤20 g (≤120 kcal) of a lipid‐based supplement, usually composed of peanuts, vegetable oil, milk powder, sugar, and added vitamins and minerals. In order to minimize disruption of IYCF practices, it is often recommended that SQ‐LNS is mixed into local complementary foods (as opposed to direct consumption; Meeting Report: Evidence and Programmatic Considerations for the Use of Small‐Quantity Lipid‐Based Nutrient Supplements for the Prevention of Malnutrition, 2016).

In the Democratic Republic of Congo (DRC), malnutrition and suboptimal IYCF practices are major public health challenges. Fewer than half of children aged 0–5 months are exclusively breastfed, and among children 6–23 months, an estimated 18% and 37% receive the WHO recommended minimum dietary diversity and meal frequency each day, respectively (DRC: DHS, 2013). Ultimately, 43% of children under 5 years are stunted and 60% of children aged 6–59 months suffer from anaemia (DRC: DHS, 2013). To address anaemia and stunting in young children, the Programme National de Nutrition (PRONANUT) of the Ministry of Public Health and the UNICEF‐DRC piloted an enhanced IYCF intervention for pregnant women and infants 0–12 months. The enhanced IYCF intervention included facility‐ and community‐based counselling on handwashing, SQ‐LNS, and IYCF, as well as the monthly distribution of SQ‐LNS for children 6–12 months.

Randomized trials have indicated that daily SQ‐LNS as part of complementary feeding can improve child growth (Adu‐Afarwuah et al., 2007; Christian et al., 2015; Hess et al., 2015; Iannotti et al., 2014) and motor development (Adu‐Afarwuah et al., 2007). An effectiveness trial in Bangladesh also showed that SQ‐LNS could be provided at scale to pregnant and lactating women and to their children (from 6 to 24 months) with ultimate improvements in linear growth among children (Dewey et al., 2017). However, there is no research on the integration of SQ‐LNS into national or subnational IYCF programmes. Integrating SQ‐LNS into IYCF programmes could contribute to increased political, financial, community, and human resources devoted to IYCF and may ultimately improve IYCF practices, as has been hypothesized about the integration of micronutrient powders (MNP) into IYCF programmes (Siekmans, Bégin, Situma, & Kupka, 2017). To date, a few integrated IYCF–MNP programmes have documented improved IYCF practices in diverse settings (Locks et al., 2017; Locks et al., 2018; Mirkovic et al., 2013). Only one study has assessed the impact of SQ‐LNS distributions on IYCF practices, and this study focused on results from randomized trials of SQ‐LNS where IYCF behaviour change was not part of the intervention (Arimond et al., 2017). Our current analysis focuses on the impact of the DRC enhanced IYCF programme (an integrated IYCF–SQ‐LNS programme) on IYCF and handwashing practices.

## 2 METHODS

### 2.1 Programme implementation and study participants

The enhanced IYCF programme was piloted in Haut‐Katanga District in Katanga Province; two health zones were selected, one as an intervention area (Kasenga) and one as a control area (Kipushi). Cross‐sectional baseline and endline surveys were conducted in both areas. The zones were selected for programmatic purposes with the intention of implementing the programme in the control area if the evaluation demonstrated an impact on childhood stunting and anaemia. The two zones were selected on the basis of the following specific criteria: (a) health areas in Haut‐Katanga District with large populations, (b) nonadjacency to Lubumbashi (the provincial capital), and (c) nonadjacency to each other. Distance from Lubumbashi was prioritized given the potential popularity of free SQ‐LNS distributions and the programmatic costs of distributions to children who resided outside of the intervention zone. Similarly, geographic distance between the intervention area and control area was essential to prevent spillover. Kasenga and Kipushi were the only two zones that fulfilled the selection criteria; however, there were some key differences in the areas. Kasenga (the intervention area) is a rural, ethnically homogenous (Bemba) area with an agricultural economy, whereas Kipushi (the control area) is a mining area that is home to several different ethnic groups.

The control area received the government IYCF strategy based on the Essential Nutrition Actions (World Health Organization, 2013), whereas the intervention area received the enhanced IYCF intervention (Table 1). Site visits and key informant interviews in both areas prior to developing the intervention protocol revealed that in both areas, counselling on IYCF was provided in some health facilities, but that the coverage and training of health workers varied substantially across facilities and the use of job aids was rare. Community health workers (CHWs) were present in both the intervention and control areas (primarily to support vaccination, vitamin A supplementation, and growth monitoring programmes); however, they generally did not provide IYCF counselling.

**Table 1:** Details of the IYCF programme in Kipushi and Kasenga, Democratic Republic of Congo

Kipushi—control area: Standard of care: Essential nutrition actions	Kasenga—intervention area: Enhanced programme that integrated IYCF, handwashing, and SQ‐LNS
Expected interventions during pregnancy: Malaria prevention (Fansidar). Daily iron–folic acid supplementation. Post‐partum vitamin A supplementation. Individual IYCF counselling from health workers during ANC visits (with limited coverage and few IEC materials).	Expected interventions during pregnancy: Malaria prevention (Fansidar). Daily iron–folic acid supplementation. Post‐partum vitamin A supplementation. Individual IYCF counselling from newly trained health workers during ANC visits with new IEC materials and logbooks to track health worker activities. Monthly home visits with individual IYCF counselling from newly trained CHW workers (with new IEC materials and logbooks to track CHW activities). Monthly CHW group meetings on IYCF (with new IEC materials and logbooks to track CHW activities).
Expected child health interventions: Vaccinations. Biannual vitamin A supplementation. Individual IYCF counselling from health workers during child health visits (with limited coverage and few IEC materials). Monthly growth monitoring (with limited coverage) at health facilities (or outreach events in remote areas) + group counselling on child health and IYCF during growth monitoring (with limited coverage and few IEC materials).	Expected child health interventions: Vaccinations. Biannual vitamin A supplementation. Individual IYCF counselling from newly trained health workers during child health visits with new information, education & communication (IEC) materials and logbooks to track health worker activities. Monthly growth monitoring at health facilities (or outreach events in remote areas) + monthly group counselling on child health and IYCF during monthly growth monitoring at health facilities or during outreach growth monitoring events in remote areas, with new IEC materials and logbooks to track health worker activities. Monthly home visits with individual IYCF counselling from newly trained CHWs (with new IEC materials and logbooks to track CHW activities). Monthly CHW group meetings on IYCF (with new IEC materials and logbooks to track CHW activities). Daily SQ‐LNS for children 6–12 months (provided to mothers monthly at the health facility).
Development of new key messages based on formative research None	Development of new key messages based on formative research: Breastfeed within 1 hr of delivery and give only breastmilk to your child from birth until 6 months without water or other food. From 6 months, continue breastfeeding and add a solid diet such as porridge enriched with eggs, insects, and fish (the animal source foods found to be available in local markets during formative research). From 6 to 12 months, mix one sachet of Kulabora (SQ‐LNS) into your child's food per day. Wash your hands with soap or ash before preparing food, eating, feeding your child, and after using the toilet.
Additional investments in the health facility platform None	Additional investments in the health facility platform: Thirty health workers participated in a 2‐day training on the enhanced IYCF programme based on UNICEF tools. Enhanced IYCF programme key messages and strategies further enforced when health workers provided 1‐day trainings for 286 CHWs (CHWs). This was also intended to strengthen the between health workers and CHWs. Key messages and implementation further enforced by the expectation that health workers provide monthly SQ‐LNS distributions to mothers of children 6–12 months (children were to receive daily SQ‐LNS from children 6–12 months). New IEC materials and programme tools on IYCF, handwashing, and SQ‐LNS (health worker guides and logbooks and leaflets).
Additional investments in the CHW platform None	Additional investments in the CHW platform: A total of 286 CHWs participated in training on enhanced IYCF programme based on UNICEF tools. CHWs received bikes to improve their mobility in their catchment areas. CHWs received new CHW guide with images on IYCF supporting the four key messages. CHWs received logbooks to track activities. Standardized expectations for the role of CHWs in the enhanced IYCF programme. Guidebooks, trainings, and logbooks standardized the expected contact points between mothers and CHWs for IYCF counselling. CHWs were expected to (a) conduct monthly home visits to pregnant women and women with children 0–12 months and (b) conduct monthly group meetings with ~20 pregnant women and women with children 0–12 months (CHWs were instructed to hold group meetings more frequently if they had a large catchment area). Improved and standardized supervision—CHWs were expected to review their logbooks each month with the CHW supervisor (a health worker at the health facility).
Investments in mass media and IEC None	Investments in mass media and IEC materials: Creation and distribution of IEC materials addressing IYCF, handwashing, and SQ‐LNS including posters (CHW and health worker guides, leaflets, and Kulabora packaging). Weekly radio messages re‐enforcing the four key messages.

*Note*. ANC: antenatal care; CHW: community health worker; IYCF: infant and young child feeding; SQ‐LNS: small‐quantity lipid‐based nutrient supplements; UNICEF: United Nations International Children's Emergency Fund.

The 2‐year enhanced IYCF programme targeted all 23,000 pregnant women and infants 0–12 months in Kasenga, using an expanded and locally adapted version of the UNICEF community‐based IYCF programme tools. The enhanced IYCF programme also included monthly distributions of SQ‐LNS for children 6–12 months and counselling for their mothers on the appropriate use of SQ‐LNS. All training and informational materials were developed on the basis of extensive formative research on the knowledge, attitudes, practices, and barriers to optimal IYCF practices. The formative research included focus groups and key informant interviews with mothers, fathers, health workers, and district officials as well as market visits to identify which nutrient‐rich foods were available during different times of the year (Tripp et al., 2015). In the intervention area, 30 health workers and 286 CHWs were trained to participate in the enhanced IYCF programme. In addition to receiving IYCF–SQ‐LNS training and counselling tools, CHWs in the intervention area were also given bikes to improve their ability to travel to remote areas and reinforce their community presence.

In the intervention area, mothers of children aged 6–12 months were expected to receive monthly distributions of SQ‐LNS from health facilities as well as community‐ and facility‐based counselling on its appropriate use. SQ‐LNS was locally branded as “*Kulabora*,” which translates to “eating better.” Monthly distributions composed of four strips of seven sachets (totalling 28); each of the seven sachets contained images and text supporting one of seven key messages on Kulabora use, which also re‐enforced key IYCF and handwashing messages: (a) one packet per child per day, (b) wash your child's hands with soap and water before feeding, (c) breastfeed your child before giving food, (d) put a small amount of food that you think your child will eat in a separate bowl, (e) mix the Kulabora into the food, (f) feed the food mixed with the Kulabora to your child, and (g) Kulabora is for children from 6 to 12 months of age.

### 2.2 Survey methodology

The baseline survey was conducted in both health zones in October 2011; the endline survey was conducted in October–November 2014. The enhanced IYCF programme was initiated in the intervention area in September 2012, with the first SQ‐LNS distributions occurring in May 2013. Both surveys employed a two‐stage cluster sampling design. Using the UNICEF Multiple Indicator Cluster Survey sampling frame, 30 clusters were selected from each health zone using probability proportional to population size. There were 72 villages with approximately 6,655 children between 6 and 17.9 months of age in Kipushi and 219 villages and approximately 4,992 children in Kasenga. Before population size sampling, large clusters were split into approximately equal segments, and small clusters were combined with nearby villages. A list of all children 6–17.9 months in each selected cluster was then developed, and 22 children from each cluster were randomly selected; there were no replacements for households who refused to participate. The intervention targeted pregnant women and infants 0–12 months; however, the surveys included children 6–18 months. Children <6 months were excluded because they would not have been exposed to the SQ‐LNS component of the programme. Children up to 18 months were included because it was hypothesized that the programme's nutritional impact would be retained for several months after the programme ended, and there were substantial cost implications of including only children 6–12 months, as sampling would have required visiting a greater proportion of households in both health zones. Sample sizes (650 children per health zone per survey) were determined for the primary outcomes of the evaluation (to provide 80% power to detect a relative decline in the prevalence of anaemia of 15%). A total of 660 households were targeted in each health zone during both the baseline and endline surveys.

In both surveys, trained interviewers asked caregivers about household sociodemographic characteristics and knowledge, attitudes, and behaviours relating to nutrition, IYCF, and SQ‐LNS. (Across both surveys >97% of caregivers were biological mothers and are thus referred to as mothers from here on). Mothers were asked if they had heard of anaemia and if so to list the causes. They were also asked to recall breastfeeding practices in early life such as whether the child had ever been breastfed, how many hours or days after birth the mother initiated breastfeeding, when the mother introduced water for the first time, and when she introduced solid and semi‐solid foods. Interviewers also asked mothers to recall all of the foods and drinks the child consumed in the previous day. Interviewers were instructed to count the number of separate meals or snacks provided to the child (to calculate meal frequency) and also to use a list of 17 food groups and mark yes or no for each food group depending on whether the food was consumed in the previous day. Because mothers did not receive SQ‐LNS before the baseline survey, dietary recalls excluded SQ‐LNS (for comparability). In the endline survey, mothers were specifically asked about SQ‐LNS receipt and children's consumption of SQ‐LNS. In the data analyses phase, the 17 food groups were collapsed to the standard seven food groups recommended in the WHO/UNICEF IYCF indicators (WHO/UNICEF/IFPRI/UCDavis/FANTA/AED/USAID, 2008). In accordance with the WHO/UNICEF indicators, minimum dietary diversity was defined as greater than or equal to four food groups (out of seven) in the previous day, minimum meal frequency as greater than or equal to two times per day for breastfed infants aged 6–8 months, greater than or equal to three times for breastfed children aged 9–23 months, and greater than or equal to four times for nonbreastfed children 6–23 months. For breastfed children, minimum acceptable diet was defined as minimum meal frequency and minimum dietary diversity. For nonbreastfed infants, minimum acceptable diet was defined as at least two milk feeds, minimum meal frequency and at least four out of six food groups (excluding the dairy food group) in the previous day. For handwashing indicators, interviewers observed whether the household had soap and then asked the mother whether she used soap the previous day and if yes, to list the purposes for which she had used soap.

In the endline survey only, mothers were asked about exposure to specific components of the enhanced IYCF programme such as the following: whether she received information about breastfeeding during her last pregnancy, whether she received information about complementary feeding or handwashing at any point, and follow‐up questions on the content and source of that information. Mothers were also asked about SQ‐LNS knowledge, attitudes, and practices, as well as general indicators of programme exposure such as whether she participated in a group session on infant feeding at her last visit to the health centre for her child, whether she knows her CHW, and whether she had heard the radio messages on IYCF.

### 2.3 Data analysis

We compared change in prevalence of key IYCF indicators from baseline to endline in the two health zones using difference in differences (DiD) analyses. Unadjusted and multivariable DiD (95% CI) estimates were obtained from mixed linear regression models with an interaction term between variables for health area (intervention vs. control) and time (endline vs. baseline), and cluster as a random effect (Card & Krueger, 1993). Based on a review of the literature, we determined a priori that multivariable models would adjust for child's sex and age; maternal age, education (completion of secondary school and completion of primary school vs. less than a primary education), and ethnicity (Bemba vs. other ethnicity); and household's primary source of income (agriculture, wage labour or daily work, or other), whether there was another child under 5 years of age in the household, and asset tertile. Asset tertile was derived from a principal component analysis of all households in the baseline and endline survey's binary yes–no responses to ownership of a radio, television, mobile phone, refrigerator, stove, chair, bed, lamp, oven, hoe, sewing machine, bicycle, car, truck, and electricity (Vyas & Kumaranayake, 2006). Analyses were first conducted stratified by child's age (among children ≤12 months and children >12 months); however, when no notable differences were found in the two age groups, they were collapsed.

Prevalence ratios comparing programme exposure at endline in the intervention and control areas were obtained from linear mixed models with a log link, binomial distribution, and cluster as a random effect (Spiegelman & Hertzmark, 2005). Within the intervention area at endline, we also compared prevalence ratios for IYCF knowledge and practices among mothers with high versus low programme exposure (defined as 2–3 vs. 0–1 of the three exposures assessed in the endline survey: attendance at a health centre group IYCF session; receipt of IYCF, SQ‐LNS, or handwashing information from a CHW; and feeding the child SQ‐LNS). The initial analysis plan was to compare mothers with any exposure to the enhanced IYCF programme (1–3 of the exposures above) to an “unexposed group”; however, only 53 mothers in the intervention area at endline answered no to all three exposures. We thus collapsed mothers with 0–1 exposures into a single reference group defined as the “low exposure” group, compared with the “high exposure” (2–3 exposures) group. When the log‐binomial models did not converge, a log‐Poisson link function, which provides a consistent but less efficient empirical estimate of the prevalence ratio (Zou, 2004), was used.

### 2.4 Ethical approval

The National Statistics Office in Lubumbashi in DRC and the U.S. Centers for Disease Control (CDC) approved the protocol for the impact evaluation; the CDC determined the evaluation as public health practice. Interviewers explained the survey protocol to mothers and also informed mothers of the option to refuse to participate in the survey or to stop participation at any point. Given low rates of literacy in the area, all mothers provided verbal, informed consent to participate in the baseline and endline surveys, and interviewers indicated on the questionnaire when informed consent had been obtained.

## 3 RESULTS

The baseline and endline surveys included 1,288 and 1,307 mothers, respectively, reflecting response rates of 97.7% and 99.2%, respectively. Intervention and control areas were similar on several key demographic characteristics such as child's age and sex, maternal age, the number of households with multiple children under 5 years, and the proportion of household who receive primary health care at health posts versus dispensaries (Table 2). However, there were several differences between the two areas; notably, over 80% of households in Kasenga (intervention area) but less than 40% of households in Kipushi (control area) cited agriculture as their primary source of income. Kipushi also had a more urban population with greater ethnic diversity, more education, and more household assets.

**Table 2:** Demographic characteristics of study participants in the baseline and endline surveys in Kipushi and Kasenga, Democratic Republic of Congo^a^

	Control area (Kipushi)		Intervention area (Kasenga)	
	Baseline *n* = 638	Endline *n* = 653	Baseline *n* = 650	Endline *n* = 654
Household characteristics				
Household location				
Urban	317 (49.7)	332 (50.8)	108 (16.6)	107 (16.4)
Rural	321 (50.3)	321 (49.2)	542 (83.4)	547 (83.6)
Primary source of income				
Agriculture	234 (36.7)	258 (39.5)	530 (81.5)	536 (82.0)
Wage labour/daily work	209 (32.8)	248 (38.0)	56 (8.6)	85 (13.0)
Other	195 (30.6)	147 (22.5)	64 (9.9)	33 (5.1)
Asset tertile^b^				
Tertile 1 (most assets)	349 (54.7)	298 (46.4)	81 (12.5)	114 (17.6)
Tertile 2	150 (23.5)	176 (27.4)	286 (44.0)	264 (40.8)
Tertile 3 (fewest assets)	139 (21.8)	169 (26.3)	283 (43.5)	269 (41.6)
Number of children under 5 years in the household				
Only the child in the survey	135 (21.2)	147 (23.3)	178 (27.4)	221 (34.1)
More than one child under 5 years	503 (78.8)	483 (76.7)	472 (72.6)	427 (65.9)
Maternal characteristics				
Mother's age				
Youngest tertile (<24 yrs)	195 (30.6)	191 (29.5)	227 (35.1)	243 (37.4)
Middle tertile (24–30 yrs)	227 (35.6)	230 (35.5)	201 (31.1)	185 (28.5)
Oldest tertile (>30 yrs)	216 (33.9)	227 (35.0)	218 (33.8)	222 (34.2)
Mother's ethnicity				
Bemba	75 (11.8)	87 (13.3)	600 (92.3)	592 (90.5)
Other^c^	563 (88.2)	566 (86.7)	50 (7.7)	62 (9.5)
Highest level of education achieved				
Secondary school or university	220 (34.5)	140 (21.4)	61 (9.4)	37 (5.7)
Primary school	309 (48.5)	330 (50.5)	329 (50.6)	168 (26.7)
Incomplete/no formal education	108 (17.0)	183 (28.0)	260 (40.0)	449 (68.7)
Maternal report of location for primary health care for the selected child				
Health post	64 (10.0)	55 (8.4)	81 (12.5)	55 (8.4)
Pharmacy	456 (71.5)	552 (84.5)	431 (66.3)	493 (75.4)
Other	118 (18.5)	46 (7.0)	138 (21.2)	106 (16.2)
Child characteristics				
Child's sex				
Male	334 (52.4)	328 (50.2)	346 (53.2)	313 (47.9)
Female	304 (47.6)	325 (49.8)	304 (46.8)	341 (52.1)
Childs age				
6.0–11.9 months	294 (46.1)	332 (50.8)	372 (57.2)	353 (54.0)
12.0–17.9 months	344 (53.9)	321 (49.2)	278 (42.8)	301 (46.0)

a All values are *n* (%)

b Based on a principle component analysis of household asset ownership including whether the household has a radio, television, mobile phone, refrigerator, stove, chair, bed, lamp, oven, hoe, sewing machine, bicycle, car, truck, and electricity.

c Other ethnicities include Kasai, Luba, Balamba, Basanga, Rund, Hemba, and Tabwa.

Compared with the control area, the intervention area saw substantial increases in the proportion of mothers who reported optimal breastfeeding practices in the first 6 months and handwashing behaviours during food and defecation‐related moments in the previous day; these differences remained significant in multivariable analyses adjusting for sociodemographic confounders (Table 3). Specifically, the proportion of mothers who reported initiating breastfeeding in the first hour after birth increased in the intervention area by 56.4% (49.3, 63.4) more than in the control area. For introducing water and complementary foods at 6 months, the multivariable DiD were +66.9% (60.6, 73.2) and +54.4% (47.6, 61.1), respectively (*P* < 0.001 for all). The increase in the proportion of mothers who reported the following activities was also greater in the intervention area compared with the control area in multivariable analyses: feeding the child the minimum meal frequency (Adj. DiD [95% CI]: +9.2% [2.7, 15.7], *P* = 0.005); fed their child in a separate bowl (+9.7% [2.2, 17.2], *P* = 0.01); awareness of anaemia (+16.9% [10.4, 23.3], *P* < 0.001); having soap in their house (+14.9% [8.3, 21.5], *P* < 0.001); and washing hands before preparing food (+12.5% [9.3, 15.6]), feeding the child (+15.0% [11.2, 18.8]), and after defecating (+10.5% [5.8, 15.2]). Notably, the significant DiD for minimum meal frequency was largely driven by a decrease in feeding frequency in the control area as opposed to an increase in the intervention area. We also did not find significant differences in the changes in the proportion of children fed the minimum dietary diversity or minimum acceptable diet in the intervention versus control area. The proportion of children receiving the minimum dietary diversity and minimum acceptable diet remained below 10% in both areas at baseline and endline, with the mean number of food groups consumed in both districts at baseline and endline of approximately two food groups only.

**Table 3:** Comparison of maternal knowledge and practices relating to infant and young child feeding (IYCF) and nutrition at baseline and endline in Kipushi (control area) and Kasenga (intervention area), Democratic Republic of Congo

	Control area (reference)		Intervention area		Unadjusted comparison		Adjusted comparison	
	Baseline^a^ (reference) *n* = 638	Endline^a^ *n* = 653	Baseline^a^ (reference) *n* = 650	Endline^a^ *n* = 654	Difference in differences % (95% CI)^b^	*P*	Difference in differences % (95% CI)^b^^,^ c	*P*
Maternal report of breastfeeding practices in the first 6 months of life								
Breastfeeding initiated within 1 hr of birth	383 (60.0)	342 (52.4)	295 (45.4)	606 (92.7)	+54.9 [48.0, 61.8]	<0.001	+56.4 [49.3, 63.4]	<0.001
Water not introduced until 6 months	123 (21.2)	122 (18.9)	64 (10.9)	489 (74.9)	+66.3 [60.2, 72.5]	<0.001	+66.9 [60.6, 73.2]	<0.001
Solid foods introduced at 6 months	177 (27.7)	155 (23.7)	170 (26.2)	505 (77.2)	+55.2 [48.6, 61.8]	<0.001	+54.4 [47.6, 61.1]	<0.001
Complementary feeding practices and nutrition knowledge								
Child was breastfed yesterday	584 (92.0)	599 (91.6)	630 (96.9)	630 (96.3)	−0.3 [−3.8, 3.3]	0.89	+1.3 [−2.2, 4.8]	0.46
Child was fed semi‐solid or solid foods previous day	624 (97.8)	625 (96.9)	620 (95.4)	609 (93.3)	−1.2 [−4.3, 1.9]	0.44	−1.6 [−4.7, 1.5]	0.32
Child was fed the minimum dietary diversity^d^	58 (9.1)	37 (5.7)	43 (6.6)	17 (2.6)	−0.6 [−4.3, 3.0]	0.73	−0.8 [−4.5, 3.0]	0.69
Child was fed the minimum meal frequency^d^	194 (30.6)	148 (23.1)	155 (23.9)	145 (22.2)	+5.9 [−0.7, 12.6]	0.08	+9.2 [2.7, 15.7]	0.005
Child was fed the minimum acceptable diet^d^	34 (5.4)	17 (2.7)	13 (2.0)	4 (0.6)	+1.3 [−1.2, 3.8]	0.30	+1.6 [−0.9, 4.1]	0.21
Child eats from own bowl	355 (55.6)	373 (57.2)	394 (60.6)	473 (72.3)	+10.2 [2.8, 17.5]	0.007	+9.7 [2.2, 17.2]	0.01
Mother has heard of anaemia	473 (74.0)	564 (86.0)	382 (58.7)	552 (84.4)	+13.7 [7.3, 20.0]	<0.001	+16.9 [10.4, 23.3]	<0.001
Mother identifies lack of iron or poor diet as a cause of anaemia	147 (23.0)	162 (24.8)	70 (10.8)	115 (17.6)	+5.1 [−0.8, 11.1]	0.09	+5.5 [−0.5, 11.6]	0.07
Handwashing behaviours								
Household has soap (observed by interviewer)	537 (84.2)	525 (80.4)	400 (61.5)	473 (72.3)	+14.7 [8.2, 21.1]	<0.001	+14.9 [8.3, 21.5]	<0.001
Mother reports using soap the previous day to:								
Wash hands after defecation	75 (11.8)	92 (14.1)	19 (2.9)	90 (13.8)	+8.5 [3.8, 13.2]	<0.001	+10.5 [5.8, 15.2]	<0.001
Wash hands before preparing food	20 (3.1)	16 (2.5)	7 (1.1)	78 (11.9)	+11.6 [8.4, 14.8]	<0.001	+12.5 [9.3, 15.6]	<0.001
Wash hands before feeding child	71 (11.1)	39 (6.0)	1 (0.2)	63 (9.6)	+14.8 [11.0, 18.5]	<0.001	+15.0 [11.2, 18.8]	<0.001

a Values are *n* (%).

b Unadjusted and adjusted difference in differences estimates were obtained from mixed linear regression models with an interaction term between health area (intervention vs. control) and time (endline vs. baseline), and cluster as a random effect.

c Multivariable models adjust for child's sex and age; maternal age, education, and ethnicity; urban versus rural; households primary source of income and household asset tertile; and whether there was another child under 5 in the household.

d In accordance with the UNICEF IYCF indicators, minimum dietary diversity is defined as four or more food groups (out of seven) in the previous 24 hr. Minimum meal frequency defined as greater than or equal to two times per day for breastfed infants aged 6–8 months, greater than or equal to three times for breastfed children aged 9–23 months, and greater than or equal to four times for nonbreastfed children 6–23 mos. Minimum acceptable diet is defined as minimum meal frequency and minimum dietary diversity in the previous 24 hr for breastfed children; for nonbreastfed children, minimum acceptable diet is defined as greater than or equal to two milk feeds, the minimum meal frequency and greater than or equal to four food groups (from a total of six food groups that excludes dairy) in the previous day.

Several programme exposure variables, such as indicators on SQ‐LNS receipt, knowledge and usage, as well as indicators on receipt of information on IYCF, were collected in the endline survey only. Analyses from the endline survey confirmed that mothers in the intervention area were substantially more likely to report exposure to programme components compared with mothers in the control area (Table 4). For example, 44.5% of mothers in the intervention area reported receiving information on breastfeeding from their CHW during pregnancy, and 44.7% and 48.3% of mothers reported receiving information from their CHW on feeding their child after 6 months and on handwashing, respectively. The comparable rates of information receipt from the CHW were respectively 0.5%, 0.3%, and 0.3% in the control area. Mothers in the intervention area were also more likely to report receiving specific messages on key IYCF behaviours including “initiate breastfeeding within one hour after birth” (adjusted prevalence ratio [APR; 95% CI]: 2.54 [1.89, 3.40]), “exclusively breastfeed for the first 6 months” (APR [95% CI]: 1.72 [1.35, 2.18]), no liquids other than breastmilk for the first 6 months (APR [95% CI]: 6.11 [3.88, 9.65]), “continue breastfeeding until at least 2 years” (APR [95% CI]: 2.02 [1.52, 2.69]), and “wash your hands before preparing food and feeding your child” (APR [95% CI]: 3.16 [2.33, 4.29] and 1.69 [1.33, 2.16], respectively). Notably, less than half of mothers in the intervention area reported receiving the key message on dietary diversity to “add eggs, insects, and fish to child's food,” and in multivariable models, there was no difference between the proportion of mothers who received this message in the intervention and control areas. Although 95.9% and 70.2% of mothers in the intervention area had heard of Kulabora (LNS) and tried feeding it to their child, respectively, only 1.4% of mothers in the control area had heard of Kulabora, and only a single mother had fed it to her child. In the intervention area, 91.3% of mothers who received SQ‐LNS reported receiving 28 sachets in their last distribution, three quarters of whom reported feeding their child all 28 sachets.

**Table 4:** Comparison of maternal exposure to the integrated infant and young child feeding (IYCF), handwashing, and small‐quantity lipid‐nutrient supplement (SQ‐LNS) intervention in the endline survey in Kipushi (control area) and Kasenga (intervention area), Democratic Republic of Congo

	Control area^a^ N = 623	Intervention area^a^ N = 654	Adjusted PR (95% CI)^b^	*P*
Breastfeeding information				
Received information on breastfeeding during last pregnancy from:				
Facility‐based health worker	488 (78.7)	586 (89.6)	1.20 [0.98, 1.46]	0.08
Community health worker	3 (0.5)	291 (44.5)	57.35 [17.1, 192.3]	<0.001
Either facility‐based or community health worker	490 (79.0)	617 (94.3)	1.23 [1.01, 1.50]	0.04
Content of information about breastfeeding received during last pregnancy:				
Initiate breastfeeding within 1 hr of birth	149 (23.9)	423 (64.7)	2.54 [1.89, 3.40]	<0.001
Exclusively breastfeed the child for 6 months	282 (45.3)	476 (72.8)	1.72 [1.35, 2.18]	<0.001
No liquids other than breastmilk in first 6 months	47 (7.5)	241 (36.9)	6.11 [3.88, 9.65]	<0.001
Complementary feeding information				
Received information about feeding child from:				
Facility‐based health worker	512 (82.7)	582 (89.1)	1.10 [0.90, 1.34]	0.29
Community health worker	2 (0.3)	292 (44.7)	Inestimable^c^	—
Either facility‐based or community health worker	512 (82.7)	614 (94.0)	1.14 [0.93, 1.38]	0.20
Content of information about feeding received:				
Add eggs, insects, and fish to child's food	382 (61.3)	285 (43.6)	0.88 [0.68, 1.13]	0.31
Breastfeed before providing other foods	97 (15.6)	350 (53.5)	3.83 [2.72, 5.41]	<0.001
Continue breastfeeding until child is at least 2 years	191 (30.7)	387 (59.2)	2.02 [1.52, 2.69]	<0.001
Information about Kulabora^d^	3 (0.5)	484 (74.0)	Inestimable^c^	—
Handwashing information				
Received information about handwashing from:				
Facility‐based health worker	542 (87.8)	579 (88.5)	1.02 [0.84, 1.24]	0.82
Community health worker	2 (0.3)	316 (48.3)	Inestimable^c^	—
Either facility‐based or community health worker	543 (88.0)	611 (93.4)	1.07 [0.88, 1.30]	0.49
Content of information about handwashing received:				
Wash your hands before preparing food	133 (21.4)	371 (56.7)	3.16 [2.33, 4.29]	<0.001
Wash your hands before feeding your child	287 (46.1)	448 (68.5)	1.69 [1.33, 2.16]	<0.001
Wash the child's hands before he/she eats	161 (25.8)	237 (36.2)	1.74 [1.26, 2.42]	<0.001
Use soap or ash with water to wash your hands	318 (51.0)	438 (67.0)	1.26 [0.99, 1.60]	0.06
Wash your hands after using the toilet	414 (66.5)	477 (72.9)	1.16 [0.93, 1.44]	0.20
Wash your hands after the child defecates	129 (20.7)	128 (19.6)	1.43 [0.94, 2.18]	0.10
Wash the child's hands after he/she defecates	61 (9.8)	63 (9.6)	1.75 [0.98, 3.13]	0.06
Lipid‐based nutrient supplement (LNS) exposure				
Mother has heard of Kulabora (LNS)	9 (1.4)	627 (95.9)	94.00 [40.79, 216.61]	<0.001
Mother has received LNS	1 (0.2)	461 (70.5)	Inestimable^c^	—
Child has tried LNS	1 (0.2)	459 (70.2)	Inestimable^c^	—
Number of times mother received LNS^e^^,^ f	—	2.3 ± 0.8	—	—
Mother received 28 sachets at the last distribution^f^	—	421 (91.3)	—	—
Child consumed all 28 sachets from last distribution^g^	—	313 (74.9)	—	—
Mean number of sachets consumed from the last distribution^e^^,^ g	—	24.7 ± 7.2	—	—
General programme indicators				
Mother participated in group session on infant feeding at her last visit to the health centre for her child	144 (23.9)	445 (69.1)	3.20 [2.39, 4.28]	<0.001
Mother knows her community health worker	348 (53.5)	608 (93.1)	1.70 [1.37, 2.11]	<0.001
Mother has seen the community health worker guide	389 (63.1)	565 (86.7)	1.53 [1.24, 1.90]	<0.001
Mother has heard the radio messages on IYCF	97 (15.2)	333 (51.1)	4.31 [2.99, 6.21]	<0.001

a Values are *n* (%) except where indicated.

b Adjusted prevalence ratios (PR) and corresponding 95% confidence intervals and *P* values were obtained from linear mixed models with a log link, binomial distribution, and a random effect for cluster. Models adjust for child's sex and age; maternal age, education, and ethnicity; urban versus rural; household's primary source of income and household asset tertile; and whether there was another child under 5 in the household. When the log‐binomial model would not converge, a Poisson distribution was used.

c Because *n* ≤ 3 mothers in the reference group had the exposure of interest, the multivariable model for the prevalence ratio cannot converge.

d Information about Kulabora includes any information about LNS including feed your child one sachet per day, mix the Kulabora with a little bit of food that your child can finish eating, and wash the child's hands before feeding him/her Kulabora.

e Values are mean ± standard deviation.

f Only among mothers who received LNS (*n* = 461 in intervention area).

g Only among children who received 28 LNS sachets in their last distribution and whose mothers reported a quantity consumed (*n* = 418 in the intervention area).

In analyses from the endline survey in the intervention area, we also found that high programme exposure (2–3 vs. 0–1 of the following: attendance at a health centre group IYCF session, receipt of IYCF or handwashing information from a CHW, and feeding the child SQ‐LNS) was associated with select IYCF and handwashing knowledge and practices (Table 5). Specifically, we found that mothers with high programme exposure were significantly more likely to report waiting until 6 months to introduce water (APR [95% CI]: 1.12 [1.04, 1.19], *P* = 0.001) and introducing solid foods at 6 months (APR [95% CI]: 1.19 [1.06, 1.32], *P* = 0.002). Mothers with high programme exposure were also significantly more likely to report feeding their child from their own bowl (APR [95% CI]: 1.10 [1.02, 1.19], *P* = 0.02) and washing their hands before feeding their child (APR [95% CI]: 2.07 [1.02, 4.19], *P* = 0.04) in the previous day than mothers with low programme exposure.

**Table 5:** Comparison of maternal knowledge and practices relating to infant and young child feeding, nutrition and handwashing in the endline survey based on programme exposure in Kasenga health zone (intervention area), Democratic Republic of Congo

	Low programme exposure^a^ (reference) *N* = 194	High programme exposure^a^ *N* = 460				
	*n* (%)	*n* (%)	Unadjusted PR (95% CI)^b^	*P*	Adjusted PR (95% CI)^b^^,^ c	*P*
Maternal report of breastfeeding practices in the first 6 months of life						
Breastfeeding initiated within 1 hr of birth	181 (93.3)	425 (92.4)	0.99 [0.94, 1.04]	0.66	0.99 [0.93, 1.06]	0.87
Water not introduced until 6 months	110 (57.0)	379 (82.4)	1.45 [1.27, 1.65]	<0.001	1.12 [1.04, 1.19]	0.001
Solid foods introduced at 6 months	128 (66.0)	377 (82.0)	1.24 [1.11, 1.38]	<0.001	1.19 [1.06, 1.32]	0.002
Complementary feeding practices and maternal nutrition knowledge						
Child was breastfed yesterday	184 (94.9)	446 (97.0)	1.02 [0.99, 1.06]	0.24	1.01 [0.90, 1.14]	0.80
Child was fed solid of semi‐solid food the previous day	175 (90.7)	434 (94.4)	1.04 [0.87, 1.24]	0.66	1.02 [0.85, 1.22]	0.83
Child was fed the minimum dietary diversity the previous day^d^	1 (0.5)	16 (3.5)	6.79 [0.90, 51.17]	0.06	Inestimable	—
Child was fed the minimum meal frequency the previous day^d^	41 (21.2)	104 (22.6)	1.06 [0.77, 1.46]	0.72	1.10 [0.82, 1.49]	0.52
Child was fed the minimum acceptable diet the previous day^d^	0 (0.0)	4 (0.9)	Inestimable	—	Inestimable	—
Child eats from own bowl	120 (61.9)	353 (76.7)	1.24 [1.10, 1.40]	<0.001	1.10 [1.02, 1.19]	0.02
Mother has heard of anaemia	151 (77.8)	401 (87.2)	1.12 [1.03, 1.22]	0.008	1.03 [0.96, 1.10]	0.36
Mother identifies lack of iron or poor diet as cause of anaemia	30 (15.5)	85 (18.5)	1.20 [0.79, 1.83]	0.40	1.27 [0.82, 1.96]	0.29
Handwashing behaviours						
Household has soap (observed by interviewer)	125 (64.4)	348 (75.7)	1.17 [1.04, 1.32]	0.009	1.07 [0.98, 1.17]	0.12
Mother reports using soap the previous day to:						
Wash hands after defecation	19 (9.8)	71 (15.4)	1.56 [0.97, 2.52]	0.07	1.53 [0.91, 2.58]	0.11
Wash hands before preparing food	20 (10.3)	58 (12.6)	1.25 [0.76, 2.05]	0.37	1.33 [0.78, 2.27]	0.29
Wash hands before feeding child	11 (5.7)	52 (11.3)	2.12 [1.10, 4.10]	0.02	2.07 [1.02, 4.19]	0.04

a Low programme exposure defined as 0 or 1 of the following, and high programme exposure is defined as at least two of the following: (a) mother attended a group session on IYCF at her last health facility visit for her child; (b) mother receiving information from her community health worker on breastfeeding, complementary feeding and/or handwashing; or (c) mother has fed her child LNS.

b Univariate and multivariate prevalence ratios (PR), 95% confidence intervals, and *P* values obtained from linear regression models with the log link and binomial distribution. When the log‐binomial model did not converge, a Poisson distribution was used.

c Multivariate models adjust for child's sex and age; maternal age, education, and ethnicity; urban versus rural; households primary source of income and household asset tertile; and whether there was another child under 5 years in the household.

d In accordance with the UNICEF IYCF indicators, minimum dietary diversity is defined as four or more food groups (out of seven) in the previous 24 hr. Minimum meal frequency defined as greater than or equal to two times per day for breastfed infants aged 6–8 months, greater than or equal to three times for breastfed children aged 9–23 months, and greater than or equal to four times for nonbreastfed children 6–23 months. Minimum acceptable diet is defined as minimum meal frequency and minimum dietary diversity in the previous 24 hr for breastfed children; for nonbreastfed children, minimum acceptable diet is defined as greater than or equal to two milk feeds, the minimum meal frequency and greater than or equal to four food groups (from a total of six food groups that excludes dairy) in the previous day.

## 4 DISCUSSION

In this analysis of cross‐sectional, preintervention, and postintervention surveys conducted in samples that were representative of all households with children aged 6–18 months in two health zones in Katanga Province, DRC, we found that the health zone that received the enhanced IYCF intervention experienced significantly greater increases in the proportion of mothers reporting several key breastfeeding and handwashing practices between baseline and endline compared with mothers in the control area. We did not, however, find significant DiD in the proportion of mothers who fed their children the minimum dietary diversity or minimum acceptable diet.

Notably, this analysis revealed that immediate and exclusive breastfeeding indicators in the first 6 months of life increased significantly more in the intervention area than the control area, and that in the intervention area, mothers with high programme exposure (including exposure to CHWs) were more likely to wait until 6 months to introduce water or complementary foods compared with mothers with low programme exposure. A separate study from South Kivu, DRC, also found that a nutrition programme using CHWs resulted in substantially higher rates of exclusive breastfeeding among children under 6 months in the intervention area compared with a control area (Balaluka et al., 2012). Taken together, these findings highlight the potential role for CHWs to improve IYCF practices in the DRC, and possibly elsewhere in sub‐Saharan Africa, when CHWs are provided appropriate training and supervision in IYCF (and in the case of the current intervention, bikes, for both transportation and an incentive). Mothers in the DRC usually only receive IYCF counselling if they attend a health facility for antenatal or child health care; however, most mothers attend health facilities infrequently—for example, less than half of mothers fulfil at least four antenatal care visits (DRC: DHS, 2013). Nationally, only 48% of children under 6 months are exclusively breastfed, and only 9% of breastfed children aged 6–23 months receive the minimum acceptable diet (DRC DHS, 2013). Expanding community‐based IYCF counselling using CHWs has the potential to contribute to improvements in nutrition knowledge, breastfeeding practices, and handwashing practices in areas with low health facility attendance.

Enhancing access to diverse, micronutrient foods is also essential for optimal IYCF practices. Although key messages of the enhanced IYCF intervention emphasized the importance of adding “special foods such as eggs, insects, and fish to the child's food,” less than half of mothers in the intervention area reported receiving this key message from their health worker. Furthermore, we found that in the baseline and endline surveys from both health zones, mothers reported feeding their children an average of only two food groups per day (below the WHO recommended four or more food groups per day). Few studies have analysed drivers of IYCF practices in the DRC; however, one qualitative study from South Kivu revealed that mothers cited the cost of meat, milk, eggs, fruits, and vegetables, as well as their lack of empowerment to make purchasing decisions, as the primary barriers to mothers providing their children a diverse diet (Burns et al., 2016). In this setting of high poverty, low maternal education and limited access to micronutrient‐rich foods (DRC DHS, 2013; Ekesa, Blomme, & Garming, 2011), health workers may not have emphasized this message if they did not think that it was feasible for mothers to improve dietary diversity, and among mothers who did receive this message, lack of access to diverse foods was likely an important barrier to behaviour change. These findings thus highlight the particular relevance of fortified foods such as SQ‐LNS to fill the micronutrient and macronutrient gap between biological needs and actual consumption of infants and young children in areas with high rates of poverty (Arimond et al., 2015).

Our study is one of few to assess the impact of a community‐ and facility‐based integrated IYCF–SQ‐LNS programme on IYCF practices (Arimond et al., 2017; Owino, Bahwere, Bisimwa, Mwangi, & Collins, 2011). One trial in South Kivu, DRC, found that moderately malnourished children randomized to a lipid‐based ready‐to‐use complementary food made from soybean, maize, and sorghum with 5% milk powder consumed the same amount of breastmilk as children randomized to the standard corn–soy blend (UNIMIX; Owino et al., 2011). A second study combined data from four coordinated randomized trials in children without malnutrition in Africa and Asia and showed there were no differences in key infant feeding practices such as continuing breastfeeding and diet diversity, and that in two sites, children randomized to receive SQ‐LNS were more likely to be fed the minimum meal frequency than children who were not randomized to LNS (Arimond et al., 2017). These findings highlight that the provision of fortified foods such as SQ‐LNS can be distributed without disrupting IYCF practices, particularly when SQ‐LNS distributions are combined with an intensive IYCF programme. Our findings indicate that integrating the counselling and distribution of SQ‐LNS with IYCF behaviour change communication may ultimately contribute to improvements in some IYCF practices. The DRC enhanced IYCF programme integrated several key messages in the behaviour change strategies for IYCF and SQ‐LNS practices. For example, the SQ‐LNS was distributed in strips of seven sachets, each with its own key message on IYCF and feeding the child SQ‐LNS. Other synergies included distributing SQ‐LNS to mothers while simultaneously providing counselling on general IYCF practices, trainings for facility‐based health workers and CHWs that included topics in IYCF and SQ‐LNS use, and the use of mass media and communications materials that integrated key messages in IYCF and SQ‐LNS use.

Our study has important limitations. Notably, our intervention and control areas, though both health zones in Haut‐Katanga District in Katanga Province, were selected for programmatic purposes and to minimize programmatic spillover. The areas were not identical in important sociodemographic characteristics. In our analyses, we adjusted for key sociodemographic characteristics such as ethnicity, maternal education, household source of income, and asset ownership; however, we cannot rule out residual confounding. In addition, our study design includes serial cross‐sectional data from representative samples of the two areas at baseline and endline; however, we did not collect longitudinal data on individual mother–infant pairs, nor did we collect programme exposure data (such as frequency of contact with health workers and CHWs) during the baseline survey. Thus, we cannot conduct longitudinal analyses to show changes in behaviour among mothers due to programme exposure. In order to support the findings that the enhanced IYCF programme played a causal role in the observed improvements in IYCF practices, we showed that mothers in the intervention area with high levels of programme exposure were more likely to engage in optimal IYCF practices compared with mothers with low programme exposure, even after adjustment for key demographic characteristics. However, we cannot rule out the possibility that there were unmeasured confounding factors that drove mothers to pursue both programme exposure and optimal IYCF practices.

In addition, our sample included children aged 6–18 months for cost reasons, whereas the programme focused on children under 12 months and pregnant women. Our findings on indicators relating to immediate and exclusive breastfeeding are based on maternal recall of at least 6 months; this recall may ultimately reflect reporting bias as opposed to actual changes in behaviour. Notably, reporting bias in this study would likely still be a reflection of changes in knowledge if not actual changes in behaviour (i.e., mothers in the intervention area would need to know that they “should” report immediate and exclusive breastfeeding in order to document the substantial increases that were recorded). Despite documented changes in maternal report of some key IYCF practices, we did not document changes in dietary diversity; and our lack of data on food prices and availability prevents us from fully understanding this lack of change. Finally, our evaluation did not collect qualitative information from mothers, CHWs, or health facility staff, and thus, we are unable to fully elucidate barriers and motivations of mothers or frontline workers.

Our study has several strengths. We collected baseline and endline data in both the programme and control areas, thus allowing us to separate the extent to which changes in IYCF practices are due to the intervention as opposed to secular trends. Our baseline and endline surveys are representative of the study areas, they collect comprehensive data on key IYCF and sociodemographic characteristics, and they included large sample sizes, all of which allowed for rigourous, multivariate analyses adjusting for key confounders. In this study, we found that there were (a) significant DiD comparing some IYCF practices at baseline and endline in the two health zones; (b) substantial differences in the proportion of mothers exposed to IYCF programme components (counselling and key messages) in the two health zones at endline; and (c) correlations between programme exposure and optimal IYCF practices within the intervention area at endline. Taken together, these findings demonstrate a consistent association between the enhanced IYCF programme and improved IYCF and handwashing practices in Kasenga, DRC.Overall, we found that the enhanced IYCF intervention was associated with an increase in the prevalence of several important handwashing and breastfeeding practices, and that mothers in the intervention area were substantially more likely to receive IYCF messages from CHWs than mothers in the control area. CHWs may be an underutilized platform for supporting IYCF programmes in the DRC and elsewhere sub‐Saharan Africa. Future research should explore cost and feasibility of activating CHWs to contribute to IYCF programmes. Notably, we did not observe a change in dietary diversity among infants in this food insecure environment. Although we cannot isolate the independent effects of SQ‐LNS in this intervention, the barriers for improving dietary diversity may highlight the particular relevance of SQ‐LNS and other fortified food supplements for increasing the consumption of micronutrients and macronutrients in areas with high rates of poverty and limited access to diverse diets. Future research should verify the potential of integrated IYCF–SQ‐LNS to improve IYCF practices, and ultimately children's nutritional status.

## CONFLICTS OF INTEREST

The authors declare that they have no conflicts of interest.

## CONTRIBUTIONS

KT, MEJ, DW, and RK designed the evaluation. KT, SN, BA, FS, and AN oversaw survey implementation. KT, SN, BA, FS, AN, MEJ, and DW provided training, supervision, and support of data collectors and provided interpretation and approval of analyses. LML and YA analysed data. LML, KT, YA, and AG wrote the paper and had primary responsibility for final content. All authors read and approved the final manuscript.
